# Using 7 cm immobilized pH gradient strips to determine levels of clinically relevant proteins in wheat grain extracts

**DOI:** 10.3389/fpls.2015.00433

**Published:** 2015-06-12

**Authors:** Sona Fekecsová, Maksym Danchenko, Lubica Uvackova, Ludovit Skultety, Martin Hajduch

**Affiliations:** ^1^Department of Developmental and Reproduction Biology, Institute of Plant Genetics and Biotechnology, Slovak Academy of SciencesNitra, Slovakia; ^2^Faculty of Natural Sciences, Comenius UniversityBratislava, Slovakia; ^3^Institute of Virology, Slovak Academy of SciencesBratislava, Slovakia

**Keywords:** *Triticum aestivum*, gel-based, quantification, MALDI-TOF/TOF, 2-DE, 7 cm IPG, grain, allergen

## Abstract

The aim of the work was to test a relatively simple proteomics approach based on phenol extraction and two-dimensional gel electrophoresis (2-DE) with 7 cm immobilized pH gradient strips for the determination of clinically relevant proteins in wheat grain. Using this approach, 157 2-DE spots were quantified in biological triplicate, out of which 55 were identified by matrix-assisted laser desorption/ionization – time of flight tandem mass spectrometry. Clinically relevant proteins associated with celiac disease, wheat dependent exercise induced anaphylaxis, baker’s asthma, and food allergy, were detected in 24 2-DE spots. However, alcohol-soluble gliadins were not detected with this approach. The comparison with a recent quantitative study suggested that gel-based and gel-free proteomics approaches are complementary for the detection and quantification of clinically relevant proteins in wheat grain.

## Introduction

The main component of the wheat grain are storage proteins with gluten as the major part representing as much as 80% of total protein content (Ferranti et al., 2007). Gluten is a mixture of gliadins and glutenins that differ in their electrophoretic mobility (Payne et al., 1985; Jacobsen et al., 2007). Gluten is also the main allergen in the wheat grain and is responsible for nutritive intolerances such as celiac disease or gluten-sensitive enteropathy (Rubio-Tapia et al., 2009), and various allergies (Battais et al., 2008; Sapone et al., 2012; Mauro Martin et al., 2014). In addition to storage proteins, wheat grain allergens include enzymatic and structural proteins such as prolamins, cupins, and Bet v1 protein family (Breiteneder and Mills, 2005). Out of these, prolamins are dominant and include α-amylase and protease inhibitors, 2S albumins, and non-specific lipid transfer proteins (nsLTPs; Breiteneder and Radauer, 2004; Mills et al., 2004).

Protein two-dimensional gel electrophoresis (2-DE) has been extensively used to characterize wheat grain proteins. For instance, 2-DE followed with immunoblotting and tandem mass spectrometry (MS/MS) resulted into the identification of nine subunits of low molecular weight (LMW) glutenins, serpin, α-amylase inhibitor, and α-gliadin in wheat flour (Akagawa et al., 2007). The combination of 2-DE and MS/MS identified several allergenic proteins, such as serpins, in dough liquor of four wheat cultivars under abiotic stress (Sancho et al., 2008). Similar approaches detected heat responsible allergenic proteins, such as α-amylase inhibitors or serpins, in the endosperm of developing wheat grains under heat stress (Hurkman et al., 2009). Additionally, several allergenic proteins were detected in Korean sprouting wheat cultivars using 2-DE and matrix assisted laser desorption/ionization-tandem Time of Flight (MALDI-TOF) MS/MS (Kamal et al., 2009). Importantly, 20 allergenic proteins in wheat grains were detected using proteomics approach based on 2-DE in combination with 17 cm immobilized pH gradient (IPG) strips and MS/MS (Yang et al., 2011). Similarly, 2-DE in combination with isoelectric focusing (IEF) capillary tube gels, three different proteases, and MS/MS resulted in the detection of 476 2-DE spots out of which 233 were identified, including well-known allergens (Dupont et al., 2011). The 2-DE was also used to analyze wheat with genetically altered omega-5 gliadin content (Altenbach et al., 2014). Interestingly, this study showed that unique genetic transformation events with the same RNA interference construct may have differential effects on the wheat grain proteome (Altenbach et al., 2014). This study highlights the importance of proteomic analyses in the study of genetic transformations (Altenbach et al., 2014).

The above studies showed that a 2-DE approach is effective in the characterization of wheat grain proteins. However, 2-DE can be labor, resources, and time consuming, especially when long IPG strips are used. The aim of this study was to test a relatively simple 2-DE approach based on 7 cm IPG strips for the detection of clinically relevant proteins of wheat grain.

## Materials and Methods

### Plant Material and Protein Extraction

Seeds of winter wheat cultivar Viginta were obtained from SELEKT LtD, Bučany, Slovak Republic. Proteins were extracted in biological triplicate from 500 mg of dry seeds. Seeds were ground in liquid nitrogen and proteins were extracted with phenol-based extraction media [50% (v/v) phenol, 0.45 M sucrose, 5 mM EDTA, 0.2% (v/v) 2-mercaptoethanol, 50 mM Tris–HCl, pH 8.8]. Sample was stirred and homogenized for 30 min at 4°C. The phenol phase was removed after centrifugation at 5000 × *g* for 10 min at 4°C. Proteins were precipitated from the phenol phase by the addition of five volumes of ice-cold 0.1 M ammonium acetate in 100% methanol, and incubated at -20°C overnight. The protein pellet was extensively washed twice using 0.1 M ice cold ammonium acetate in 100% methanol, followed by 80% ice cold acetone, and finally with 70% ice cold ethanol and precipitates were collected by centrifugation for 15 min., 5000 ×*g* at 4°C. Total protein concentration was determined using the Bradford (1976) assay with Bovine Serum Albumin as the standard.

### Two-Dimensional Gel Electrophoresis

Samples (50 μg protein) were diluted in 100 μl of IEF buffer [8 M urea, 2 M thiourea, 2% (w/v) CHAPS, 2% (v/v) Triton X-100, 50 mM dithiothreitol], 3 μl of ampholytes were added, and loaded onto 7 cm IPG strips of pH 3–10 (ReadyStrip^TM^ IPG Strips BioRad) for IEF. Isoelectric focusing was carried out using Protean IEF Cell (Bio-Rad) with the following conditions: 150 V for 150 VH, 500 V for 500 VH, and 4000 V for 15,000 VH including initial active rehydration for 12 h at 50 V. For the second dimension (SDS-PAGE), IPG strips were incubated in SDS equilibration buffer [1.5 M Tris-HCl pH 6.8, 6 M urea, 30% (v/v) glycerol, 5% (w/v) SDS) for 15 min with 2% (w/v) dithiothreitol] followed by a second equilibration step of 15 min with the equilibration buffer containing 2.5% (w/v) iodoacetamide. The equilibrated strips were loaded on the top of 10% polyacrylamide gel and the electrophoresis was run at 80 V until the dye reached the bottom of the gel. Gels were stained for 16 h with Coomassie Brilliant Blue G-250 at room temperature. The 2-DE gels were digitalized using a GS-800 Calibrated Densitometer (Bio-Rad) at 300 dpi and 16 bit grayscale. Digitalized gels were analyzed with PDQuest 8.0 software (Bio-Rad).

### Protein Digestion and Mass Spectrometry

Excised 2-DE plugs were washed with 300 μl destaining solution (50% acetonitrile in 50 mM ammonium bicarbonate) and dehydrated in 100% acetonitrile. After removal of acetonitrile, 2-DE spots were rehydrated with trypsin (Promega) and digested at 37°C overnight. The digestion was stopped with formic acid and extracted tryptic peptides were stored at -80°C until MS/MS analysis with a TOF/TOF mass spectrometer in combination with MALDI using an ultrafleXtreme instrument equipped with a 355 nm smartbeam-2 laser, capable of pulsing frequency 1 kHz (Bruker). Peptides were concentrated to 20 μl using Concentrator plus (Eppendorf). After that, concentrated peptides were desalted by μ-C18 ZipTips (Merck Millipore). Next, 1 μl of purified digests were spotted onto 800 μm AnchorChip MALDI target (Bruker) and α-cyano-4-hydroxycinnamic acid (CHCA) matrix (0.7 mg⋅ml^-1^ in 85% acetonitrile, 0.1% trifluoroacetic acid, 1 mM ammonium phosphate) was added.

The mass spectrometer was operated by flexControl 3.3 software (Bruker). For every position 4000 shots were summed in positive reflector mode in the range of 700–3500 mass to charge (m/z). Following that, up to 25 of the most intense precursor peaks per sample were selected for the MS/MS analysis with the minimal signal to noise (S/N) ratio set to 15. Abundant trypsin and keratin peaks were specified in the exclusion list. Fragmentation spectra were acquired by accumulation of 3000 laser shots in positive reflector LIFT mode. Fragmentation was achieved by laser induced dissociation (LID) mechanism by 50% increase in laser power, without the introduction of a collision gas. Simultaneously detector voltage was boosted by 80%.

### Processing of MS/MS Data

Acquired spectra were processed by flexAnalysis 3.3 software (Bruker). A sophisticated numerical annotation procedure (SNAP) algorithm was used for peak picking to calculate exact monoisotopic masses. For the precursor spectra the S/N threshold was set to 10 and the resulting spectra were externally recalibrated against data from an adjacent spot containing nine peptides of the Peptide calibration standard 2 (Bruker). For the fragment spectra S/N threshold was set to 5, also baseline subtraction (TopHat algorithm), and smoothing (Savitzky-Golay algorithm 3 cycles with 0.15 m/z width) were applied.

The MS/MS peak lists were imported into the ProteinScape 2.1 proteomic data management software (Bruker). Peptide identification was performed by an in-house Mascot 2.3 server (Matrix Science), querying against the non-redundant Triticeae plant protein UniProt database downloaded on April, 2014 (100 981 entries). Additionally, protein assignments were verified by searches against the SwissProt database from June 2014 (545 388 sequences) that included major contaminants such as trypsin or keratin. Search parameters were the following: fixed cysteine carbamidomethylation, variable methionine oxidation, one missed trypsin cleavage site, 40 ppm precursor mass tolerance, 0.5 Da fragment mass tolerance. Protein identifications were accepted if at least two different matched peptides had ion score higher than 30, meaning *p* < 0.05.

For allergenicity assessments, identified proteins were queried against the Allergome database^1^ containing 2994 allergen entries, using Allergome Aligner module with an embedded NCBI blastp v.2.2.18 algorithm. Only hits with 100% sequence identity were accepted as clinically relevant allergens. The MS/MS proteomics data have been deposited to the ProteomeXchange Consortium via the PRIDE partner repository with the dataset identifier PXD002067.

## Results

**FIGURE 1:**
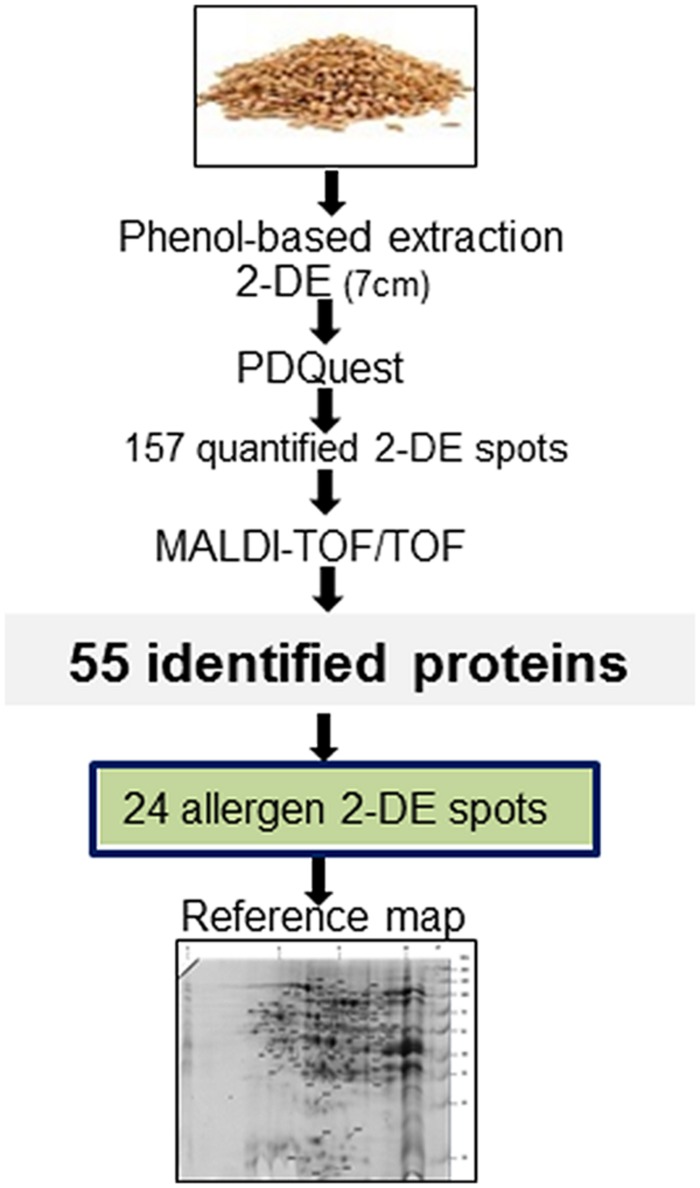
**Experimental workflow.** Proteins were isolated from the winter wheat variety Viginta using a phenol-based method and resolved by 7 cm immobilized pH gradient (IPG) strip. After analysis with PDQuest, two-dimensional gel electrophoresis (2-DE) spots were excised from the gels, and analyzed by MALDI-TOF/TOF. From 55 identified 2-DE spots, 24 contained clinically relevant proteins. These proteins were assigned onto a 2-DE reference map.

**FIGURE 2:**
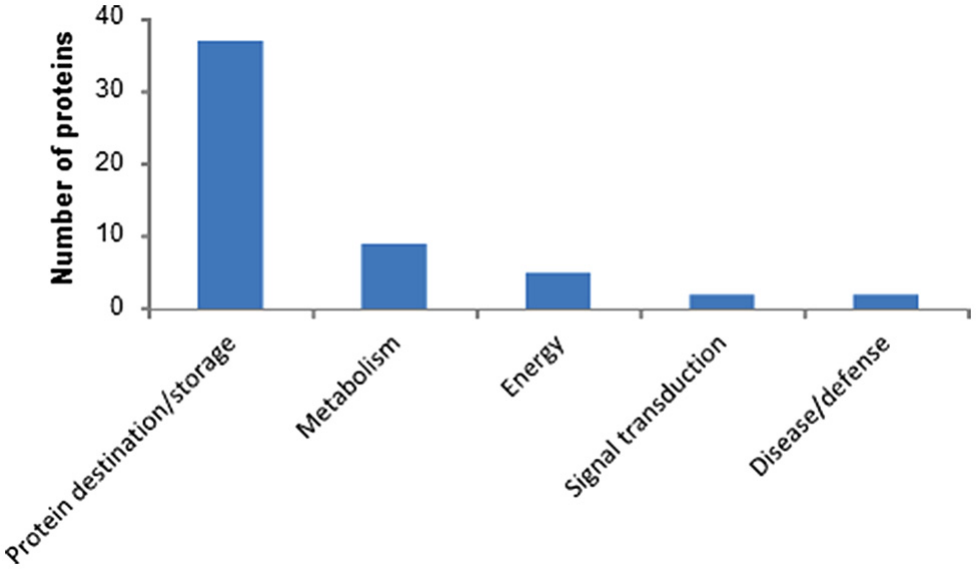
**The 55 identified wheat grain proteins were classified into five functional categories**.

The 2-DE-based proteomics approach in combination with 7 cm IPG strips (**Figure 1**) quantified 157 2-DE spots in biological triplicate (Supplementary Figure S1; Supplementary Table S1) out of which 55 were identified (**Table 1**). Identified proteins were classified according to previous apporaches (Bevan et al., 1998) into five functional classes (**Figure 2**). The most abundant class was 37 proteins associated with destination and storage, followed by nine proteins associated with metabolism and five energy proteins (**Figure 2**). This study also detected two signaling proteins and two proteins associated with disease/defense (**Table 1**). All identified proteins were assigned on the 2-DE gel (Supplementary Figure S2). The most abundant protein on this reference map is the high molecular weight (HMW) glutenin subunit (GS) with a relative volume (%V) of 8.8 (spot number 2909) followed by 11-S seed storage domain containing protein (3302) with %V of 5.6 (**Table 1**).

**Table 1:** The list of 55 identified and quantified proteins in wheat grain extracts.

Protein description	Accession number	Spot number	%V ± SD	Theor. MW/pI	Exp. MW/pI	Score	Pep./Cov.	Allergome code
**01 Metabolism**		
*01.05 Sugars and polysacharides*		
Endogenous alpha-amylase/subtilisin inhibitor	P16347	5004	0.24 ± 0.15	19.6/6.9	15.5/8.5	327.6	7/49	9593
Alpha-amylase/trypsin inhibitor CM3	P17314	4002	0.25 ± 0.15	18.2/9.1	10.0/7.7	286.9	6/43	1051
Alpha-amylase inhibitor 0.19	P01085	4009	0.08 ± 0.03	13.3/7.6	10.0/7.9	223.7	4/35	8186
Beta-amylase	W5EKI0	523	0.17 ± 0.20	61.1/4.9	71.1/5.6	376.6	7/14	**`_**
Beta-amylase	W5EKI0	1501	2.19 ± 1.16	61.1/4.9	36.1/5.9	543.5	8/20	**`_**
Beta-amylase	W5EKI0	601	0.32 ± 0.28	61.1/4.9	76.7/5.3	736.5	11/29	**`_**
Aldo/keto reductase domain containing protein	W5A6D0	4102	1.27 ± 1.41	35.9/6.7	37.6/7.8	157.2	4/13	**`_**
Glucose/ribitol dehydrogenase	W5AB71	3101	0.67 ± 0.27	31.8/6.3	37.2/7.4	107.4	3/15	**`_**
Glucose/ribitol dehydrogenase	W5AB71	3103	1.06 ± 0.17	31.8/6.3	36.5/7.6	160.8	3/15	**`_**
**02 Energy**		
*02.01 Glycolysis*		
Glyceraldehyde-3-phosphate dehydrogenase	C7C4X1	3203	0.21 ± 0.11	36.5/6.8	45.2/7.4	172.7	4/19	9502
Glyceraldehyde-3-phosphate dehydrogenase	C7C4X1	3216	0.04 ± 0.03	36.5/6.8	45.1/7.2	168.6	4/19	9502
Glyceraldehyde- 3-phosphate dehydrogenase	W5HB91	5203	0.39 ± 0.14	38.4/7.8	44.5/8.1	488.0	6/20	**`_**
Enolase domain containing protein	W5FPI0	1403	0.50 ± 0.64	49.1/5.2	60.7/6.1	74.4	2/8	**`_**
*02.10 TCA pathway*								
Malic enzyme	A0A077S3A3	2503	1.13 ± 0.85	64.0/6.1	72.0/7.1	178.0	4/9	**`_**
**06 Protein destination and storage**		
*06.13 Proteolysis*		
Serpin domain containing protein	W5I5X1	304	0.10 ± 0.05	43.4/5.0	49.9/5.4	97.8	2/9	**`_**
Serpin-Z2B	P93692	307	0.20 ± 0.11	43.0/5.1	51.8/5.6	229.2	4/14	5724
Serpin-Z2B	P93692	2202	0.04 ± 0.04	43.0/5.1	48.2/6.6	92.8	2/6	5724
Serpin-Z1A	Q41593	1203	0.13 ± 0.05	43.1/5.5	48.2/6.1	298.6	4/20	5724
Serpin-Z1A	Q41593	1204	0.41 ± 0.11	43.1/5.5	47.8/6.3	277.6	3/13	5724
Serpin-Z1B	P93693	1301	0.42 ± 0.23	43.0/5.3	51.9/5.9	271.4	4/19	5724
Serpin-Z1B	P93693	1302	0.44 ± 0.14	43.0/5.3	51.4/6.1	170.9	2/10	5724
Serpin-Z1C	Q9ST58	1303	0.36 ± 0.05	42.9/5.6	51.3/6.4	224.3	3/16	5724
*06.20 Storage proteins*		
Cupin 1 domain containing protein	W5ECA4	3004	1.07 ± 0.4	69.4/6.6	15.2/7.4	127.5	3/4	**`_**
Cupin 1 domain containing protein	W5EST8	4602	0.17 ± 0.03	70.6/6.7	81.8/7.7	346.4	7/13	**`_**
Cupin 1 domain containing protein	W5EST8	4604	0.25 ± 0.09	70.6/6.7	80.8/7.8	231.0	7/15	**`_**
Cupin 1 domain containing protein	W5ECA4	4606	0.26 ± 0.07	69.4/6.6	80.1/8.0	192.8	4/8	**`_**
Cupin 1 domain containing protein	W5ECA4	4610	0.19 ± 0.14	69.4/6.6	78.2/8.0	94.1	3/7	**`_**
Cupin 1 domain containing protein	W5EST8	5401	0.32 ± 0.31	70.6/6.7	57.4/8.2	224.6	5/10	**`_**
Cupin 1 domain containing protein	W5EAP7	5601	0.36 ± 0.10	68.9/7.4	76.8/8.1	218.2	6/14	**`_**
Cupin 1 domain containing protein	W5EAP7	5607	0.43 ± 0.18	68.9/7.4	76.2/8.5	294.9	6/14	**`_**
Globulin-3A	I6QQ39	6403	0.83 ± 0.18	66.3/9.4	57.9/8.7	257.7	6/15	**`_**
Globulin-3A	I6QQ39	6602	0.21 ± 0.06	66.3/9.4	75.4/8.5	290.4	6/13	**`_**
Globulin-3A	I6QQ39	6605	0.29 ± 0.17	66.3/9.4	75.5/8.8	610.5	14/31	**`_**
Globulin-3A	I6QQ39	7401	0.35 ± 0.27	66.3/9.4	57.5/9.0	136.3	5/13	**`_**
Globulin-3A	I6QQ39	7605	0.37 ± 0.15	66.3/9.4	75.2/9.2	405.3	9/23	**`_**
Globulin-3A	I6QQ39	8401	0.49 ± 0.24	66.3/9.4	57.7/9.4	407.9	7/18	**`_**
Globulin-3A	I6QQ39	8402	0.31 ± 0.18	66.3/9.4	57.7/9.5	226.6	5/11	**`_**
11-S seed storage domein containing protein	W5AKY9	3302	5.58 ± 3.41	64.9/6.5	52.4/7.4	556.2	6/13	**`_**
11-S seed storage domein containing protein	W5AKY9	3304	1.06 ± 0.75	64.9/6.5	51.0/7.6	343.6	5/9	**`_**
High molecular weight glutenin subunit PW212	P10389	1905	0.26 ± 0.34	89.1/5.8	128.2/6.3	150.0	3/5	2898
High molecular weight glutenin subunit PW212	P10389	1907	0.31 ± 0.37	89.1/5.8	130.4/6.4	196.8	3/5	2898
High molecular weight glutenin subunit PW212	P10389	2908	0.15 ± 0.07	89.1/5.8	159.2/7.0	78.8	3/3	2898
High molecular weight glutenin subunit DX5	P10388	2909	8.78 ± 6.72	90.2/6.5	120.0/7.0	663.6	12/11	2898
High molecular weight glutenin subunit DX5	P10388	3902	0.69 ± 0.20	90.2/6.5	120.2/7.4	285.3	8/5	2898
High molecular weight glutenin subunit DY10	P10387	5612	1.77 ± 1.86	69.6/8.5	90.2/8.5	726.9	12/14	2898
High molecular weight glutenin subunit DY10	P10387	7702	0.53 ± 0.20	69.6/8.5	90.6/9.0	130.0	3/5	2898
High molecular weight glutenin subunit 12	P08488	5702	3.67 ± 2.77	70.8/8.5	90.1/8.1	473.4	7/7	2898
High molecular weight glutenin subunit 12	P08488	4701	0.87 ± 0.28	70.8/8.5	92.2/7.9	387.1	9/7	2898
Low molecular weight glutenin subunit 1D1	P10386	7211	4.46 ± 2.60	34.9/10.5	33.4/9.1	174.0	4/8	2674
Low molecular weight glutenin subunit 1D1	P10386	7309	0.31 ± 0.33	34.9/10.5	27.3/11.0	151.6	4/8	2674
Avenin-like b7	D0EWS4	7103	0.21 ± 0.21	32.3/9.4	35.0/9.0	148.5	3/8	**`_**
**10 Signal transduction**		
Ricin B lectin domain containing protein	W5DYH0	3102	0.94 ± 1.04	39.3/6.4	38.6/7.5	357.8	6/21	**`_**
Purple acid phosphatase	W5HKQ5	2402	0.11 ± 0.02	52.9/6.3	57.7/7.2	129.9	3/10	**`_**
**11 Disease/defense**		
*11.06 Detoxification*		
Peroxidase	W5DLG4	7209	1.62 ± 0.97	38.9/9.1	40.0/9.6	133.3	4/15	**`_**
Cys peroxiredoxin PER1	Q6W8Q2	3002	0.30 ± 0.07	24.0/6.1	25.9/7.4	165.9	2/14	9499

*The table shows protein name, accession number, spot number, the (two-dimensional gel electrophoresis) 2-DE spot number assigned by PDQuest, %V ± SD, relative volume ± SD from biological triplicate analysis (Supplementary Table S1); Theor. MW/pI, theoretical molecular weight / pI; Exp. MW/pI, experimental molecular weight / pI from two-DE gels; Score, ProteinScape score; Pep./Cov., number of peptides matched to the protein sequence/ sequence coverage; Allergome code, the reference for the allergen after sequence query against the Allergome database.*

### As Much as 45% of Identified Proteins were Associated with Various Allergies or Food Intolerances

**FIGURE 3:**
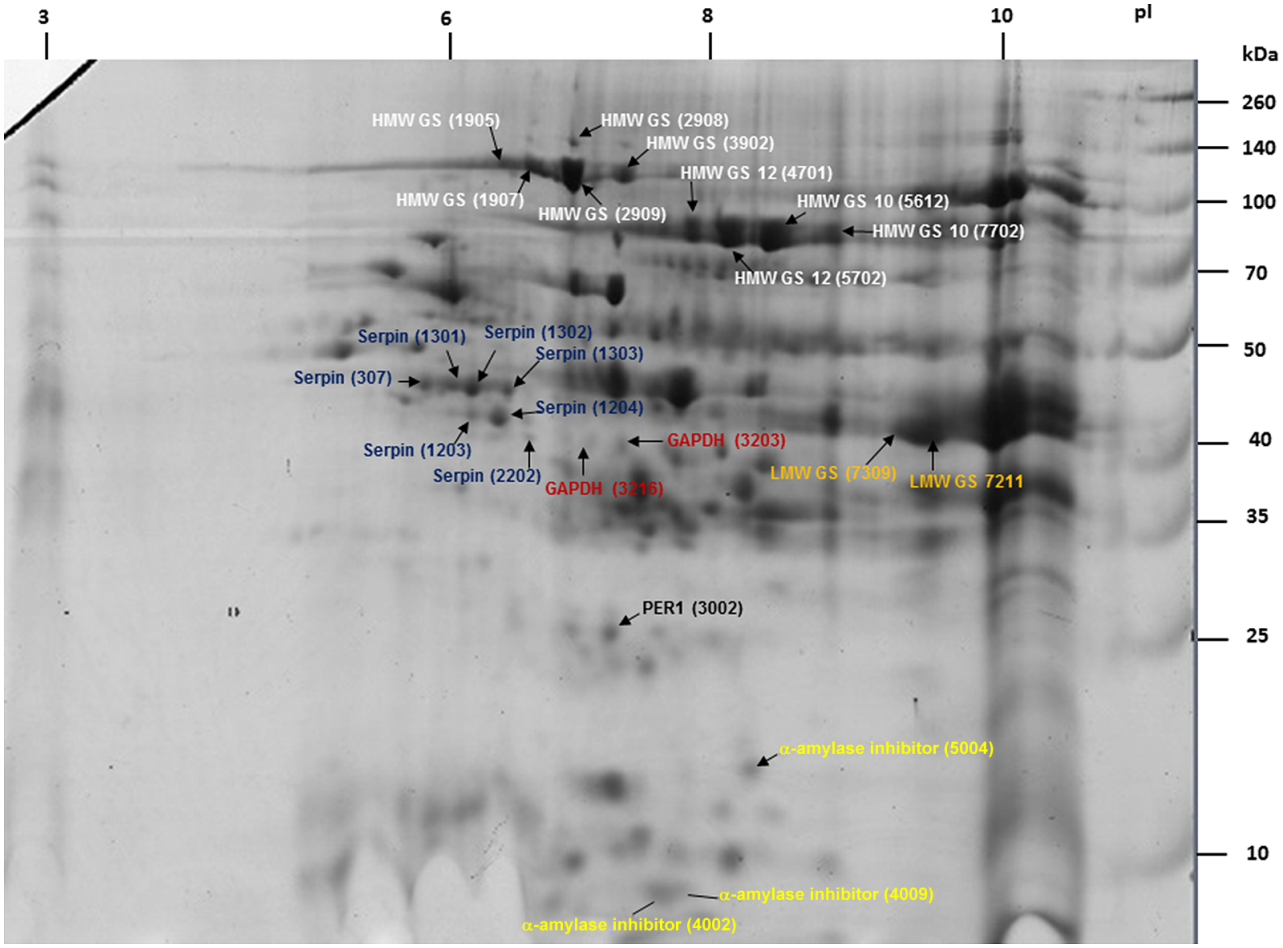
**The 2-DE reference map of 24 clinically relevant proteins representing 13 unique accession numbers (six proteins)**.

**FIGURE 4:**
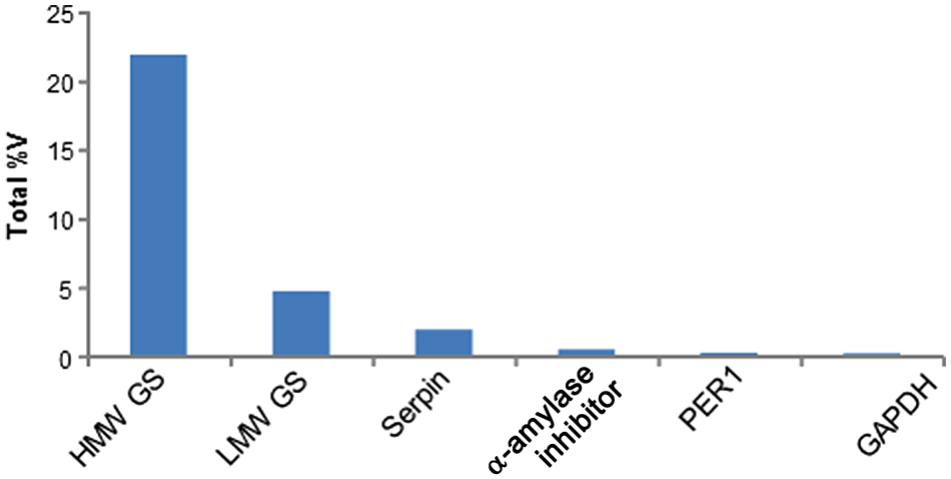
**Summed abundance of the six non-redundant allergenic proteins detected in wheat grain extracts**. The total abundance is shown as relative volume (%V).

To determine clinical relevance of the identified proteins, sequences were queried against the Allergome database^1^ which contains 2994 allergen entries (Supplementary Table S2). This approach detected clinically relevant proteins in 24 2-DE spots which represented 13 non-redundant accession numbers (**Table 1**). Out of these, nine 2-DE spots were identified as HMW GS, seven as serpins, three as α-amylase inhibitors, two as LMW GS, two as glyceraldehyde-3-phosphate dehydrogenase (GAPDH), and one as Cys peroxiredoxin (PER1; **Table 1**). All these proteins were assigned on the 2-DE gel in order to establish the reference map of clinically relevant proteins of wheat grain (**Figure 3**). All 24 2-DE spots presented on this reference map were color-coded based on protein identification to visualize regions of the 2-DE gel with a prevalence of clinically relevant proteins (**Figure 3**). The most abundant protein is HMW GS (2-DE spot number 2909) followed LMW GS (7211; **Table 1**). The 2-DE spots identified as serpin (2202), GAPDH (3216), GAPDH, and α-amylase inhibitor (4009) (3203) showed the lowest abundance on this reference map (**Figure 3**; **Table 1**). To reveal overall abundances of the detected clinically relevant proteins, relative volumes of each 2-DE spot were summed based on protein identifications. Using this approach, the summed (total) relative volumes for each of eight detected clinically relevant proteins was established (**Figure 4**). It was revealed that HMW GS, LMW GS, and serpins are highly abundant in wheat grain (**Figure 4**).

## Discussion

The aim of this work was to test a 2-DE proteomics approach with 7 cm IPG strips and phenol-based protein extraction for the detection of clinically relevant proteins in wheat grain. Classical methods for protein isolation from wheat grain are based on iso-propanol extraction (van den Broeck et al., 2009). We successfully implemented this method and have previously determined quantities of wheat grain proteins using gel-free proteomics approach (Uvackova et al., 2013a,b). In the present study we tested the phenol-based extraction protocol (Hurkman and Tanaka, 1986), which also solubilizes membrane proteins often excluded from alcohol-based protein extractions. Previously, our group efficiently used this protocol for the characterization of seed proteins in soybean (Hajduch et al., 2005; Danchenko et al., 2009; Klubicova et al., 2012), canola (Hajduch et al., 2006), castor (Houston et al., 2009), *Arabidopsis* (Hajduch et al., 2010), and flax (Klubicova et al., 2010, 2013).

In the present study we detected nine 2-DE spots as HMW GS (**Table 1**; **Figure 3**), which influence the viscoelastic properties of wheat flour (Masci et al., 1998), and may cause wheat dependent exercise-induced anaphylaxis (WDEIA) when digested (Hofmann et al., 2012). The present study was particularly successful in the detection of wheat grain allergens associated with Baker’s asthma (Salcedo et al., 2011; Olivieri et al., 2013). Five 2-DE spots were identified as serpin (**Table 1**; **Figure 3**), which are involved in food allergy and Baker’s asthma (Salcedo et al., 2011; Mameri et al., 2012). Three 2-DE spots were identified as an α-amylase inhibitor, important contributors to Baker’s asthma (Tatham and Shewry, 2008; Salcedo et al., 2011), food allergies (James et al., 1997), and WDEIA (Hofmann et al., 2012). Additionally, one 2-DE spot was detected as PER1 which is a confirmed wheat allergen likely associated with Baker’s asthma (Pahr et al., 2012). However, this study did not detect the 27 kDa albumin, which was shown to be associated with Baker’s asthma (Weiss et al., 1993) or the alcohol-soluble gliadin proteins involved in celiac disease (Wieser, 1996; Allred and Ritter, 2010).

The majority of wheat grain allergenic proteins detected in the present study were not quantified in our recent MS-based study (Uvackova et al., 2013b). This finding is in agreement with a recent investigation of soybean under flooding stress, where only 9 out of 115 proteins were detected by both gel-based and gel-free proteomics approaches in root tips (Yin et al., 2014). Similar results have been shown in the analysis of the honey bees hemolymph proteome, where only 27% of proteins were detected with both approaches (Bogaerts et al., 2009).

Based on this, it is tempting to speculate that gel-based and gel-free approaches are complementary for the detection and quantification of wheat grain allergenic proteins. However, the complementarity of gel-based and gel-free proteomics approaches was suggested previously (Luque-Garcia et al., 2011; Abdallah et al., 2012). The combination of gel-based and gel-free proteomics was shown to be effective for the analyses of soybean under flooding (Yin et al., 2014), phytopathogenic fungus *Botrytis cinerea* (Gonzalez-Fernandez et al., 2013), *Nicotiana tabacum* trichomes (Van Cutsem et al., 2011), the honeybee hemolymph proteome (Bogaerts et al., 2009), or during soybean seed filling (Agrawal et al., 2008).

## Conclusion

This study has demonstrated that phenol-based protein extraction in combination with 2-DE and 7 cm IPG strips is capable of determining clinically relevant proteins in wheat grain extracts. However, important clinically relevant proteins, such as alcohol-soluble gliadins were not detected with this approach. The comparison of these data with previous work suggests that gel-based and gel-free proteomics are complementary approaches for the determination of clinically relevant proteins in wheat grain extracts.

## Conflict of Interest Statement

The authors declare that the research was conducted in the absence of any commercial or financial relationships that could be construed as a potential conflict of interest.

## Footnotes

1 http://www.allergome.org/
