# Supplementary figures and images for: Using 7 cm immobilized pH gradient strips to determine levels of clinically relevant proteins in wheat grain extracts
Source: Front Plant Sci. 2015 Jun 12;6:433. doi: 10.3389/fpls.2015.00433 (PMC4464201; doi:10.3389/fpls.2015.00433)

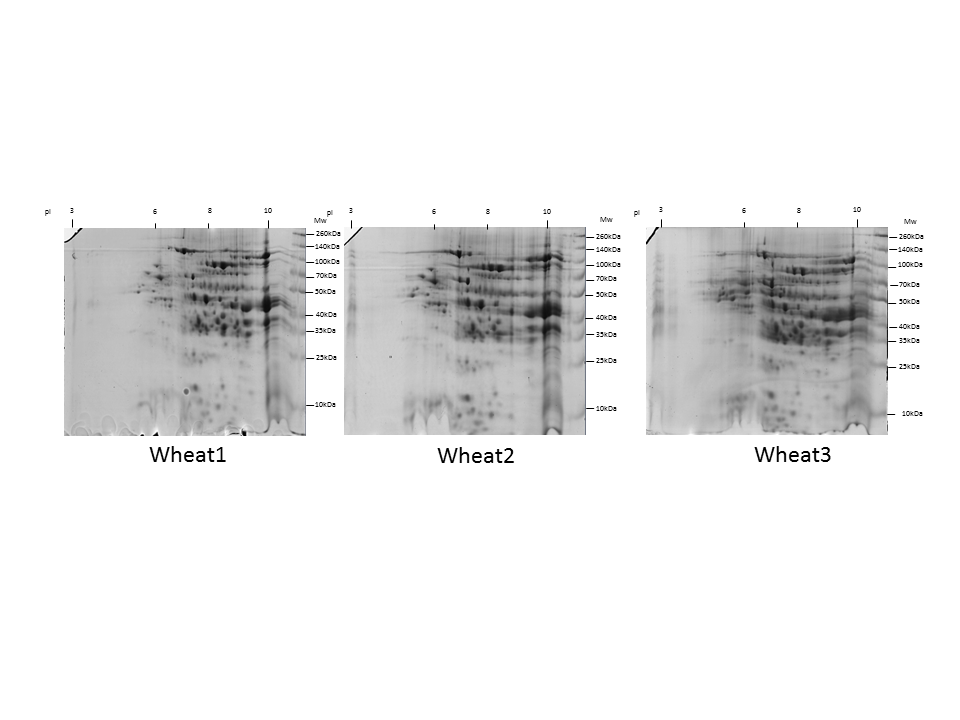

Supplement: Supplementary file 3 [file Supplemental_Figure_1.TIF]

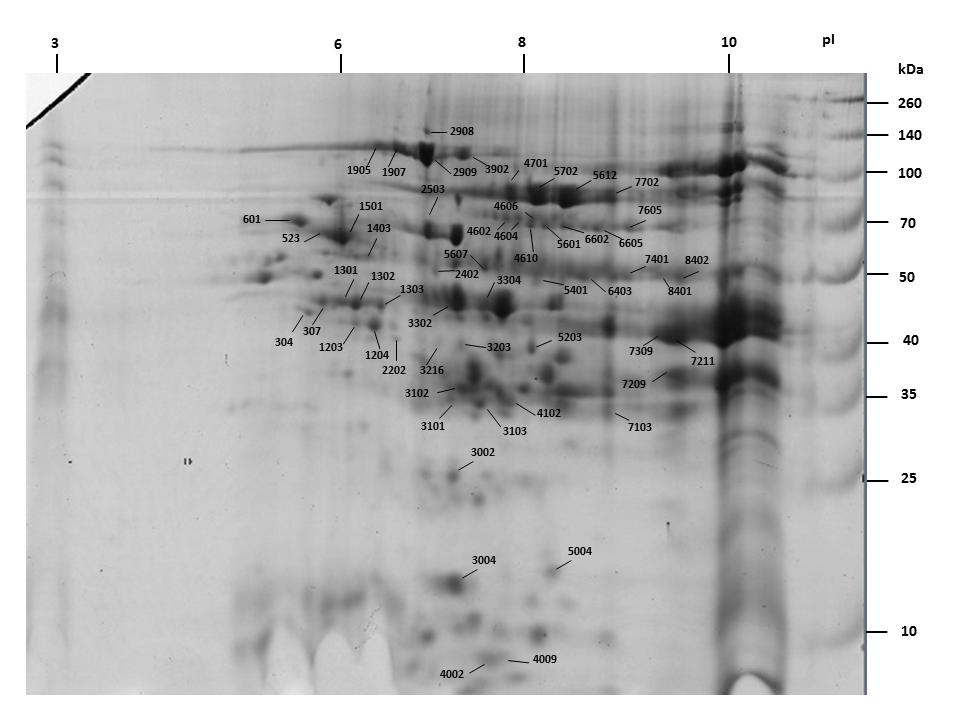

Supplement: Supplementary file 4 [file Supplemental_Figure_2.TIF]
